# Primary health care use from the perspective of gender and morbidity burden

**DOI:** 10.1186/s12905-014-0145-2

**Published:** 2014-11-30

**Authors:** María Teresa Carretero, Amaia Calderón-Larrañaga, Beatriz Poblador-Plou, Alexandra Prados-Torres

**Affiliations:** University of Zaragoza, Zaragoza, Spain; EpiChron Research Group on Chronic Diseases, Aragón Health Sciences Institute (IACS), IIS Aragón, Miguel Servet University Hospital, Zaragoza, Spain; Red de Investigación en Servicios de Salud en Enfermedades Crónicas (REDISSEC), Carlos III Health Institute, Madrid, Spain; Grupo de Investigación en Servicios Sanitarios (GRISSA), Aragón Health Sciences Institute (IACS), IIS Aragón, Zaragoza, Spain

**Keywords:** Morbidity burden, Gender, Primary health care

## Abstract

**Background:**

Sex and gender can interact to contribute to differences in morbidity and mortality between men and women. To detect such differences is an important issue for health policy planners when designing programmes for the provision of healthcare services for the whole population. Our aim was to study differences between men and women in the use of Primary Health Care (PHC) resources, taking into account age and morbidity burden.

**Methods:**

An observational retrospective study was carried out using the information gathered in electronic medical records from 79,809 adult patients who attended a PHC centre at least once in 2008. The ACG® System was used to quantify the morbidity burden of patients. Poisson regression models were applied to analyse differences in the number of visits to the PHC centre by men and women.

**Results:**

Morbidity burden was significantly higher in women of all age groups. The gross number of visits to the PHC centre was also higher for women in all age groups. However, when adjusting by age and morbidity burden, we did not find a higher utilization by women compared to men. For high levels of morbidity burden, the attendance by men was even significantly higher.

**Conclusions:**

The overall higher use of PHC by women seems to be associated with their higher morbidity burden. The interaction between biology and socially constructed roles could also underlie this higher use by women, and is therefore an area that deserves further in-depth research.

## Background

Women and men have biological differences (sex) that may result in different health risks and needs. In addition to biological factors, they also differ in social roles and responsibilities (gender) that may have implications for differences in health status and health behaviour, as well as in access and use of health services [1]. Sex and gender can interact to contribute to differences in morbidity and mortality between men and women. To detect such differences is an important issue for health policy planners when designing programmes for the provision of healthcare services for the whole population [2].

Many studies have analysed the combination of social and biological causes of the differences in the health of men and women [3-6]. Research undertaken in various countries has often shown that the use of healthcare services is higher among women [7-10], and also that women generate a higher health cost [11]. However, studies about these differences in utilization of services mainly refer to patients with particular diseases [12,13], to people of particular age ranges, such as the elderly [14,15], or to different ethnic groups [16]. Very few articles analyse these differences in a broad age range or take into account the morbidity burden [17,18].

In a context of important demographic and epidemiological changes, multimorbidity is increasingly becoming the rule among patients attending health services. The morbidity burden is a key determinant for the utilization of Primary Health Care (PHC) services and, therefore, is a variable that should be included in studies examining the use of such services [19]. Within PHC, any information provided by such studies would be very useful when designing programmes for the management of patients with multiple chronic diseases, a very important current issue in many countries [19].

In general, studies about the health of men and women are based on health surveys or on self-declared data. Although these sources provide useful information [20], self-appraisal of medical conditions may differ from the real and objective health status of the individuals [21].

The aim of the present work was to analyse the differences in the number of visits to PHC between men and women in the adult population, taking into account their morbidity burden. The study was carried out using information based on electronic medical records, which offer an excellent opportunity to study the type and burden of morbidity in population subgroups at individual level [22].

## Methods

An observational retrospective study was carried out using the information from electronic medical records gathered at seven urban PHC Centres in Aragón, Spain. The studied population included all patients over 14 years of age who visited their PHC Centre at least once in 2008. This represented around 60% of the total population assigned to the participating PHC centres. Health Centres were previously selected based on criteria related to the quality of the collected information [23].

For each patient, information was obtained for the following variables: demographic (age and sex); diagnoses (based on the International Classification of Primary Care ICPC-2); and utilization of PHC services. This study was approved by the Ethics Committee of Clinical Research of Aragón (CEICA, for its initials in Spanish), which waived the need for written patient consent because the study was based on the statistical analysis of anonymous data.

The morbidity burden was measured using the number of Ambulatory Diagnosis Groups (ADG) provided by the Johns Hopkins Adjusted Clinical Groups (ACG) System. This system assigns all ICPC-2 diagnoses registered in the medical records during one year to one of 32 non-mutually exclusive clusters or ADGs according to duration (acute, recurrent, or chronic), severity (e.g., minor and stable versus major and unstable), diagnostic certainty (symptoms versus documented disease), aetiology (infectious, injury, or other) and specialty care involvement (e.g., medical, surgical, obstetric) of diseases [24]. Thus, each ADG is a grouping of diagnoses similar in terms of both clinical criteria and expected need for healthcare resources. Just as individuals may have multiple diagnosis codes, they may have multiple ADGs (up to 32). To avoid using groups with very few individuals, those with 7 or more ADGs were grouped into one single category.

The utilization of resources was measured by the number of visits to the PHC centre. A visit was defined as only face-to-face contact of the patient with the health team (GP and/or nurse), when attending the PHC centre.

To make managing age easier, 3 cut-off points were established according to age distribution: closer value to the mean minus 1 Standard Deviation (SD); the mean; and the mean plus 1 SD. The resulting groups were the following: ≤30 years; 31-50 years; 51-70 years; and >70 years.

The mean and 95% confidence intervals were calculated for continuous variables as well as the frequency distribution for categorical variables; to find out the possible differences by gender on the number of visits to the PHC centre the Mann-Whitney U test was carried out, with the level of significance set at 5%. Moreover, to analyse differences in the number of visits to the PHC by men and women, adjusting by age and morbidity burden, a multivariable Poisson regression model was applied including a scale parameter to account for over-dispersion of the outcome variable.

All statistical analyses were undertaken using the SPSS program version 15.0.

## Results

The study population included 79,089 patients over 14 years of age. There were 44,602 (56.4%) women in the sample; in all age groups the number of women was significantly higher than that of men.

**Figure 1:**
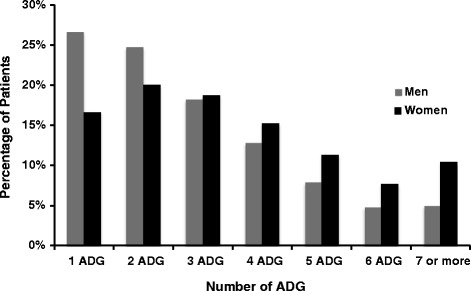
**Percentage of patients according to the number of ADGs.**

Figure 1 shows that there was a higher percentage of men with one and two ADGs. For a morbidity burden of three ADGs, the percentage was similar for both men and women. However, the percentage of women was higher for a morbidity burden of four or more ADGs.

**Figure 2:**
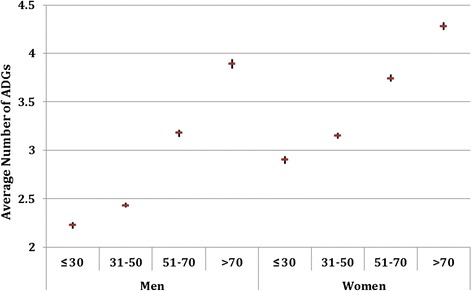
**Average number of ADGs and 95% CI by age group.**

As seen in Figure 2, the morbidity burden increased with age for both men and women. In all age groups, the morbidity burden was significantly higher for women compared to men (p < 0.05).

**Figure 3:**
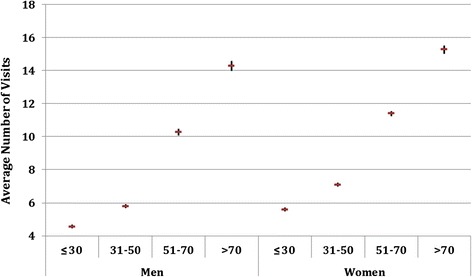
**Average number of annual visits and 95% CI by age group.**

**Figure 4:**
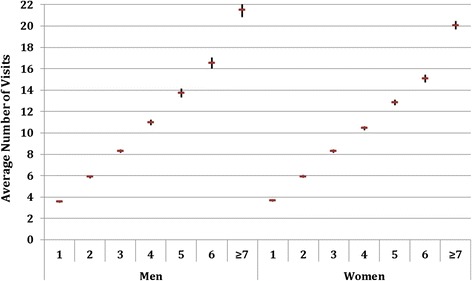
**Average number of annual visits and 95% CI by morbidity burden.**

When looking at the total number of visits to PHC centres by age group (Figure 3), women had a higher attendance in all age groups (p < 0.05). However, when analysing the global number of visits taking into account the morbidity burden (Figure 4), figures were very similar in men and women for a burden below 4 ADGs. Yet, above that level (4 or more ADGs) the number of visits was higher for men (p < 0.05).

Table 1 shows the average number of visits to PHC for the different age groups of men and women, taking into account their morbidity burden. The number of visits to PHC increased in all age groups with the number of ADGs. In most age and morbidity burden groups, the number of visits was similar between men and women. For the second age group (31-50), and for those with a morbidity burden of 6 and 7 or more ADGs, the number of visits was significantly higher for men compared to women, both from a statistical and clinical viewpoint.

**Table 1:** **Average number of annual visits and 95% CI of men and women by age group and morbidity burden**

	≤**30 Years**				**31-50 Years**				**51-70 Years**				**>70 Years**			
**No ADG**	**Male (n = 7173)**		**Female (n = 8732)**		**Male (n = 11314)**		**Female (n = 14159)**		**Male (n = 10364)**		**Female (n = 12973)**		**Male (n = 5636)**		**Female (n = 8738)**	
	**%**	**No visits**	**%**	**No visits**	**%**	**No visits**	**%**	**No visits**	**%**	**No visits**	**%**	**No visits**	**%**	**No visits**	**%**	**No visits**
1	37.6	2.53*	24.4	2.61*	34.3	2.98*	20.5	2.94*	19.4	4.93	12.9	4.84	10.4	7.30	8.0	7.39
		(2.43 -2.63)		(2.49 –2.73)		(2.87 – 3.09)		(2.82 – 3.06)		(4.70 – 5.17)		(4.59 – 5.09)		(6.77–7.83)		(6.86–7.92)
2	29	4.02	24.5	3.99	27.1	4.69*	23.1	4.76*	23.2	7.56	17.7	7.50	17.0	9.69	13.7	9.62
		(3.87 -4.18)		(3.84 –4.13)		(4.52 – 4.86)		(4.61 – 4.92)		(7.28 – 7.85)		(7.24 – 7.75)		(9.22 –10.15)		(9.16–10.08)
3	17.6	5.69	19.5	5.53	17.5	6.70	19.5	6.63	19.3	9.53*	19.0	9.68*	18.5	12.06	16.3	12.29
		(5.41 -5.97)		(5.32 –5.73)		(6.42 – 6.97)		(6.41 – 6.86)		(9.19 – 9.87)		(9.40 –9.97)		(11.58 -12.55)		(11.75–12.83)
4	8.9	7.55	13.8	7.17	10.4	8.63	14.2	8.64	15.6	12.13	16.6	11.71	17.5	14.21	16.4	13.84
		(7.04 -8.06)		(6.88 –7.47)		(8.18 – 9.07)		(8.35 – 8.94)		(11.65-12.60)		(11.38 –12.03)		(13.61 -14.08)		(13.37–14.31)
5	3.9	9.72	8.3	8.98	5.7	10.98	9.9	10.34	10.1	14.52	12.6	13.77	13.7	16.50	14.8	16.64
		(8.73 -10.70)		(8.51 –9.45)		(10.24-11.72)		(9.93 – 10.75)		(13.86-15.19)		(13.34 –14.20)		(15.64 –17.36)		(16.01–17.26)
6	1.8	11.78	5.0	10.80	2.7	13.41*	6.2	11.79*	6.4	16.70	8.5	15.82	9.9	19.14	11.6	18.97
		(10.49 -13.07)		(10.14 –11.46)		(12.28-14.53)		(11.23 –12.35)		(15.87-17.53)		(15.27 –16.37)		(18.12 -20.16)		(18.14–19.79)
≥7	1.2	14.75	4.4	13.97	2.3	19.64*	6.7	16.70*	6.2	21.29	12.6	20.43	13.1	23.06	19.2	23.01
		(12.63 -16.87)		(13.13 –14.82)		(17.84-21.45)		(15.92-17.48)		(20.32-22.26)		(19.84 –21.02)		(22.01 -24.11)		(22.34–23.68)

n: number of individuals; in brackets 95% CI.

*p < 0.05, using Mann-Whitney U test.

In the multivariable Poisson regression model, sex was no longer significantly associated to PHC utilization (IRR 0.997 95% CI 0.987-1.007) when age (IRR 1.005 95% CI 1.005-1.006) and morbidity burden (IRR 1.248 95% CI 1.245-1.252) were considered.

## Discussion

This study shows that women have a significantly higher burden of morbidity than men in all age groups. Our overall results also indicate that women visit PHC centres more frequently than men. However, when those figures were analysed by age group and morbidity burden, and adjusted accordingly, we did not find a higher utilization of PHC services by women compared to men. Indeed, although the multivariable regression model did not show any significant association between sex and PHC service use, for high levels of morbidity burden, and hence increased clinical complexity, the attendance by men was significantly higher.

Regarding morbidity burden, Uijen and van de Lisdonk [25] and Salisbury et al. [26] also found that women had a higher morbidity burden than men, although in the latter study the differences were not as high as ours, even though they also used the ACG System for measuring morbidity. Our results slightly differ from those published by Fortin et al. [27] in a study in Canada showing a higher morbidity burden for men than for women, but only for a particular age group and using a different system for quantifying the number of health problems. In Ireland, a more recent study found that multimorbidity was not affected by gender [28], but the study was restricted to people over 50 years of age. The higher morbidity burden in women is frequently explained by the difference in biology and gender; the interaction between biology and socially constructed roles is not often taken into account [1].

For PHC utilization, we found that the number of visits clearly increased with morbidity burden and that women went significantly more often to PHC centres than men. The higher attendance by women has been previously reported [7-11] and, in general, a higher use of health resources by women has been suggested [10,15,16,29,30]. Yet, those studies did not take into consideration that, in general, women have a greater morbidity burden than men, which seems to explain these differences in PHC use.

The results of an interesting recent study by Wang et al [18] can be compared to those of our study. Although they found that crude primary consultation rate -every consultation between a health professional and a patient- was 32% lower in men than women, when underlying morbidities were comparable in men and women they reported that consultation rates were similar.

In general, the higher utilization of health services by women found in many studies has been associated to a worse perception of their own health [6,8]. Social reasons, their role as carer of the family health or having a protective attitude towards disease, could also be suggested. Our data show that the morbidity burden of women is higher than that of men, i.e. although women may have a worse perception of their health, it is clear that they objectively have a worse health than men. Yet, in our study, when considering morbidity burden, differences in utilization of PHC services were not significantly higher for women and in some groups even higher for men, as in the group of middle-aged very ill patients. This could be explained because for women looking after the health of the rest of the family may be at the expense of their own health. Another reason could be that in many cases women are able to use more effective coping strategies, such as seeking social support, in particular that from other females in response to stress [31]. Therefore, the need for professional health consultation may be reduced for certain medical conditions. Nevertheless, women are more likely than men to visit a GP when a problem is serious; a close relationship between consultation behaviour and severity of the problem has been found in women but not in men [32].

Our work has two main strengths. First, the use of data gathered from electronic medical records has been suggested to be the most appropriate way for undertaking this type of study [28]. Second, the ADG system has been used to measure the morbidity burden. This system is considered to overcome medical coding problems and to allow an appropriate evaluation of the morbidity burden of individuals from PHC [33].

The possible limitations of this study are that, being based on medical records, it could be underestimating the morbidity burden of the population, as there could be patients who do not visit the PHC centres and, therefore, would not be identified [26]. Moreover, the relationship between morbidity burden and the number of visits may carry the risk of creating a vicious circle, in which people going more often to the centres are more likely to have more diseases diagnosed. Nevertheless, electronic medical records, as compared to administrative claims, offer greater accuracy of patients’ morbidity burden, because there is no reimbursement linked to data registration. Thus, new episodes of care are not necessarily generated for every visit to the GP. Finally, in this study the socioeconomic characteristics of the population have not been analysed, a factor considered to be important regarding the morbidity burden and the use of health services.

## Conclusions

The overall higher use of PHC by women seems to be associated with their higher morbidity burden. The interaction between biology and socially constructed roles could also underlie this higher use by women, and is therefore an area that deserves further in-depth research.
